# Role of αENaC in root resorption of adjacent teeth due to entirely impacted mandibular third molars

**DOI:** 10.1186/s12903-024-04040-z

**Published:** 2024-03-21

**Authors:** Jiaqi Tang, Weijun Yu, Lu Lin, Ruhan Yang, Guanglong Li, Min Jin, Yuting Gu, Bin Jiang, Eryi Lu

**Affiliations:** Department of Stomatology, Renji Hospital, Shanghai Jiao Tong University School of Medicine, 160 Pujian Road, Shanghai, 200127 China

**Keywords:** Impacted teeth, External root resorption, Epithelium sodium channel, Inflammation, Epithelial-mesenchymal transition

## Abstract

**Background:**

Entirely impacted mandibular third molar (EIM3M) concerns the pathological external root resorption (ERR) of the adjacent mandibular second molar (M2M) and formation of granulation tissue between two molars. The study aimed to clarify the effect of αENaC, a mechano-sensitive molecule, to explore the mechanical mechanism in this scenario.

**Methods:**

The force EIM3M exerted on M2M was proved by finite element analysis. αENaC expressions were tested by real-time polymerase chain reaction (PCR), immunoblotting and immunofluorescence. Inflammatory and epithelial-mesenchymal transition (EMT)-related molecules expressions were also detected by real-time PCR. The correlation was analyzed by Spearman’s correlation analysis, and receiver-operator characteristic (ROC) curve was further exhibited.

**Results:**

The force was concentrated in the ERR area. αENaC was upregulated, positively correlated with ERR degree and localized to the fibroblasts in ERR granulation tissues. Moreover, αENaC was respectively and positively associated with elevated TNF-α and N-cadherin in ERR granulation tissues. More importantly, ROC analysis verified αENaC as a novel indication of the incidence of this disease.

**Conclusions:**

Our finding revealed the force from EIM3M causing ERR of M2M, and elucidated the expression and localization of αENaC and its positive correlation with inflammation, EMT and disease severity, suggesting a novel indication in this disease.

**Supplementary Information:**

The online version contains supplementary material available at 10.1186/s12903-024-04040-z.

## Background

Long-term impacted teeth can be related to some pathological conditions, such as root resorption of the adjacent tooth and marginal bone loss [1, 2]. Studies have demonstrated that the prevalence of external root resorption (ERR) in second molars may overcome 20% in samples involving the maxillary and mandibular third molars, rising to 50% if only mandibular third molars were involved [3, 4]. Clinically, it is spotted that many entirely impacted mandibular third molars (EIM3Ms) defined as fully bone-covered here result in resorption of the distal root of mandibular second molars (M2Ms). The shadow of granulation tissues between the two molars, and ERR of adjacent M2Ms was observed by radiography. The reason for ERR of M2Ms and formation of granulation tissues is worth exploring. We speculated that this phenomenon relevant to EIM3Ms might be caused by the force exerted by EIM3Ms on the distal root of M2Ms. Therefore, we would like to explain the phenomenon from a mechanical point of view.

Particular attention is given to epithelium sodium channel (ENaC) which consists of three homologous subunits (alpha, beta and gamma) that allow Na + ions to flow through high-resistance epithelial cells and maintain salt and water homeostasis in the body [5–7]. Quantitative analyses show that ENaC activity is mediated through the pore-forming αENaC, the most abundant subunit [8–10]. The channel opening probability (Po) of ENaC is regulated by proteases, mechanical forces and shear stress [11], and laminar shear stress directly activates ENaC activity by increasing ion channel Po [12]. Hence, ENaC is considered mechano-sensitive and its mechano-sensitivity represents an additional non-drug-dependent regulatory mechanism in the regulation of ENaC activity [12]. Besides, αENaC has something to do with proliferation and migration of cancer cells through epithelial-mesenchymal transition (EMT) in ovarian cancer and then predicts poor prognosis [13], but its effect related to EMT on inflammation has not been investigated.

Epithelial-mesenchymal transition (EMT) is essential in development, wound healing and stem cell behavior [14]. We speculated that EMT might play a crucial part in the process of transdifferentiation of epithelial rests of Malassez (ERMs) in the periodontium of the distal root of M2Ms to fibroblasts in the ERR granulation tissues in EIM3Ms. In view of this hypothesis, we intended to investigate the role of αENaC in ERR of adjacent M2Ms due to the force exerted by EIM3Ms.

Mechanical force-induced ERR is usually studied in orthodontic tooth movement [15–17], and many scholars have demonstrated that tumor necrosis factor (TNF) -α played an important role in root resorption under the compressive force [18, 19]. Thus, the correlation between αENaC and TNF-α in the inflammatory root resorption of M2Ms needed in-depth study.

All above, we have searched for a new scene in which an EIM3M exerts a force on the distal root of an adjacent M2M and results in ERR. From a mechanical point of view, the force was visualized by finite element analysis (FEA), and ERR granulation tissues were collected in this scene to investigate αENaC from aspects of its upregulated expression, localization to fibroblasts and correlation with the inflammation factor (TNF-α) and the EMT-related factor (N-cadherin). Moreover, the ERR degree of M2Ms was quantified to explore the correlation between αENaC and disease severity. More importantly, we carried out the ROC analysis to substantiate that αENaC might be a potential indication of the incidence of this disease, hoping to regard αENaC as a target regulator of ERR prevention.

## Materials and methods

### Human sample collection

The demographic information was collected by interviewers, and the clinical characteristics were obtained by the ERR degree analysis (Table 1).

**Table 1:** Demographic characteristics and clinical characteristics of individuals with normal soft tissues and patients with ERR granulation tissues

	Normal tissues (*n* = 30)	Granulation tissues (*n* = 30)
Age(y)		
Mean ± SD	27.70 ± 4.16	28.63 ± 5.36
Range	20–38	21–41
Gender (male/female)	13/17	14/16
ERR degree (mm^3^)	ns	4.99 ± 4.47

Data are expressed as mean ± SD

The study was performed at Department of Stomatology, Renji Hospital Affiliated to Shanghai Jiao Tong University School of Medicine, following the STROBE guidelines (Supplementary File 1). This study protocol was approved by the Ethics Committee of Renji Hospital Affiliated to Shanghai Jiao Tong University School of Medicine (KY2021–196-B) and was performed in accordance with the Declaration of Helsinki. Informed consent was sought from each participant.

Statistically, the prevalence of impacted third molars in adults is 13.7% [20], and the prevalence of ERR in second molars may overcome 20%. As a result, the sample size was obtained according to the following formula as 60, and the sample size of the experimental and the control groups was 30 separately.

$$n=\frac{z_{\alpha}^2\ast p\ast \left(1-p\right)}{\delta^2}$$

Tissue samples were collected at Department of Stomatology, Renji Hospital Affiliated to Shanghai Jiao Tong University School of Medicine. The surgeon conducted the full thickness flap operation on the buccal side, used a surgical hand-piece for bone removal and teeth separation, removed the teeth fragment with vascular clamp, scraped and collected the granulation tissues with vascular clamp and spoon excavator, and finally sewed up the wound. The normal soft tissues were collected from freshly extracted teeth. All the soft tissues were soon stored at − 80 °C without any medium before analysis.

For soft tissues in controlled group, normal tissues located subalveolar were obtained from 30 individuals who accepted extraction of nonfunctioning third molars or orthodontic teeth, with no abnormalities on the cone-beam tomography images. For soft tissues in experimental group, ERR granulation tissues between the two molars were collected from 30 individuals who accepted extraction of EIM3Ms with ERR of adjacent M2Ms, with the cone-beam tomography images which showed EIM3Ms and the ERR shadows of adjacent M2Ms.

All the participants engaged in this study were aged from 18 to 50, and without a history of trauma or temporomandibular joint lesions or jaw lesions, orthodontic treatment, long-term medication use, cancer or undergoing radiotherapy, pregnant, lactating, or menopausal status, or any systemic disease, antibiotic use in the last 3 weeks, or chronic infectious disease during the examination. The collected samples from 60 participants were used in this study.

### Finite element analysis (FEA)

A three-dimensional (3D) reconstruction model was created from a cone-beam tomography image (i-CAT Imaging Sciences International, Hatfield, Pennsylvania) belonging to Department of Stomatology, Renji Hospital Affiliated to Shanghai Jiao Tong University School of Medicine. Here, we used one case to generate the FEA for visualization of the clinical scenario, without statistical analysis.

The cone-beam tomography data were then imported into an interactive medical imaging software (Mimics 18.0, Materialise Dental, Leuven, Belgium). Different structures (teeth, soft tissues and bone) were segmented through image density thresholding. Periodontal ligament layers (0.2 mm thick) were exerted on teeth roots by Boolean operations.

The finite element mesh was generated on Ansys v.16 software (Ansys Inc., Canonsburg, PA, USA) through the STL surface models exported from segmented structures. The mechanical properties (Young’s modulus and Poisson’s ratio) for each structure (enamel, dentin, cortical bone, cancellous bone and periodontal ligament) were determined according to the literature. Based on data obtained from previous studies, the structures were characterized as linear, elastic, and isotropic. Stresses were recorded in all structures.

### ERR degree analysis

We obtained the cone-beam tomography images (i-CAT Imaging Sciences International) of all the patients. Then, we used the measurement tool of the software to first locate the most severe ERR in the sagittal position of M2Ms and measure the vertical axis (unit: mm). Two horizontal semi-axis (unit: mm) were obtained in the coronal and transverse positions according to the location. They were measured twice in the same position to detect the intra-class correlation coefficient (ICC), and the ICC values were 0.988 for the vertical axis, 0.909 for the horizontal semi-axis in the coronal position and 0.928 for the horizontal semi-axis in the transverse position. We approximated the resorption pit as a half of an ellipsoid to obtain the ERR degree (unit: mm^3^).

### Real-time polymerase chain reaction (PCR)

Total RNA of tissues was leached with TRIzol (Invitrogen, Carlsbad, California, USA), and reverse-transcribed into cDNA with PrimeScript™ RT Master Mix (Takara Bio, Otsu, Shiga, Japan). The mRNA levels were measured by real-time PCR using FastStart Universal SYBR Green Master Mix (Roche, Nutley, NJ, USA) according to the manufacture’s instruction on a QuantStudio7 Flex Real-Time PCR System (Applied Biosystems, Foster City, CA, USA). Relative mRNA expression levels of detected αENaC, TNF-α, N-cadherin were normalized to the β-actin mRNA level. Primers were synthesized by Sangon Biotech (Shanghai, China), and were (human) as follows: αENaC, forward, 5′-GCTGATAACCAGGACAAAACACAA-3′; reverse, 5′-CGTCGCTGGGGCAGGAA-3′; N-cadherin, forward, 5′-TGCGGTACAGTGTAACTGGG-3′; reverse, 5′-GAAACCGGGCTATCTGCTCG-3′; TNF-α, forward, 5′-TCTGGGCAGGTCTACTTTGG-3′; reverse, 5′-GGTTGAGGGTGTCTGAAGGA-3′; β-actin, forward, 5′-CATGTACGTTGCTATCCAGGC-3′; reverse, 5′-CTCCTTAATGTCACGCACGAT-3′.

### Immunoblotting

Protein in ERR granulation tissues in EIM3Ms and normal tissues located subalveolar was lysed in RIPA Lysis Buffer (Beyotime, Shanghai, China) containing 1% PMSF (Beyotime) for 30 min on ice. The Thermo Scientific Pierce BCA Protein Assay Kit (Thermo Fisher Scientific, Waltham, MA, USA) was used for measuring concentration of the released protein. Protein samples were diluted, denatured, separated in SDS-polyacrylamide gels with equal amounts and transferred onto polyvinylidene fluoride membranes (Millipore, Billerica, MA, USA). The blots were cut prior to hybridization with antibodies according to the molecular size provided by merchants. After blocked with 5% BSA, membranes were exposed to 5% BSA with primary antibodies against β-actin (Cell Signaling Technology, Danvers, MA, USA) and αENaC (Thermo Fisher Scientific) overnight at 4 °C, and then incubated with the HRP-conjugated secondary antibody (Cell Signaling Technology) at room temperature for 1 hour. Immunoreactive bands were visualized by chemiluminescence reagents (Millipore).

### Immunofluorescence

The ERR granulation tissues were frozen and sliced, and then dried at room temperature. After washed with PBS, these cells were fixed with 4% paraformaldehyde, permeabilized with 0.2% Triton X-100, blocked with 5% bovine serum albumin (BSA), and then incubated with primary antibodies overnight at 4 °C. Primary antibodies included αENaC (Thermo Fisher Scientific), and Fibronectin (Thermo Fisher Scientific). The secondary antibodies were respectively Cy3-labeled Goat Anti-Rabbit IgG (Servicebio, Wuhan, China) and Alexa Flour-labeled Goat Anti-Mouse IgG (Servicebio). Nuclei were counterstained with DAPI (Cell Signaling Technology). Finally, the slides were imaged using a fluorescence microscope (Echo Revolve, San Diego, CA, USA) or a laser scanning confocal microscope (Leica-SP8, Wetzlar, Germany), and the colocalization of αENaC with Fibronectin was observed.

### Statistical analysis

All measurement data were statistically analyzed with GraphPad Prism software (version 9) and presented as mean ± SEM. Statistical significance was evaluated by Mann-Whitney U test. Correlations were analyzed according to Spearman’s correlation analysis. Significance was expressed as: *p < 0.05, ** *p* < 0.01, *** *p* < 0.001 and **** *p* < 0.0001.

## Results

### Visualization of the clinical scenario by FEA

**Fig. 1:**
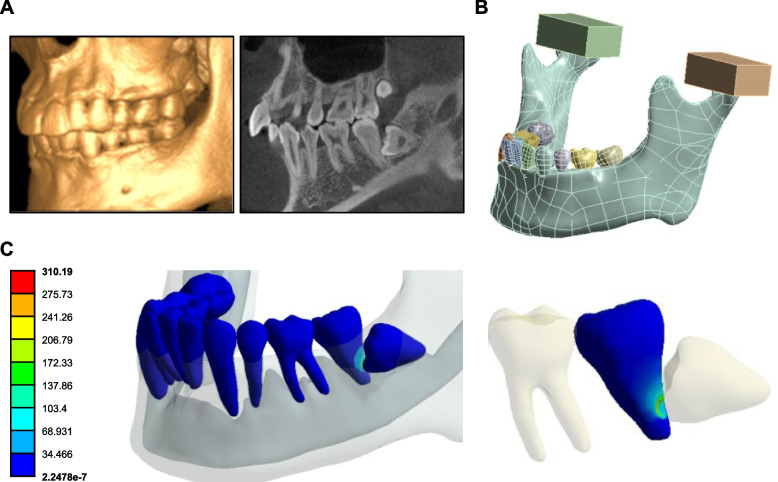
The ERR of M2M was caused by the force that EIM3M exerted on the distal root of M2M. **A** The cone-beam tomography images of one selected participant were displayed by i-CATVision. **B** The three-dimensional (3D) reconstruction model was created from the cone-beam tomography data. **C** Equivalent (von Mises) stress, unit: MPa. The area of high energy dissipation was observed in the contact region between the EIM3M and the M2M

A 3D reconstruction model was created from a cone-beam tomography image by an interactive medical imaging software (Fig. 1A, B). Analysis of the equivalent von Mises stress showed areas of high energy dissipation in the contact region between the EIM3M and the M2M. In the ERR area of M2M, the highest von Mises stress was 310.19 MPa (Fig. 1C). Besides, the von Mises stress of this area was significantly higher than those of other teeth and the M2M except ERR (Fig. 1C). Hence, ERR of M2Ms was caused presumably by the force that EIM3Ms exerted on the distal root of M2Ms.

### Upregulation of αENaC expression in ERR granulation tissues

**Fig. 2:**
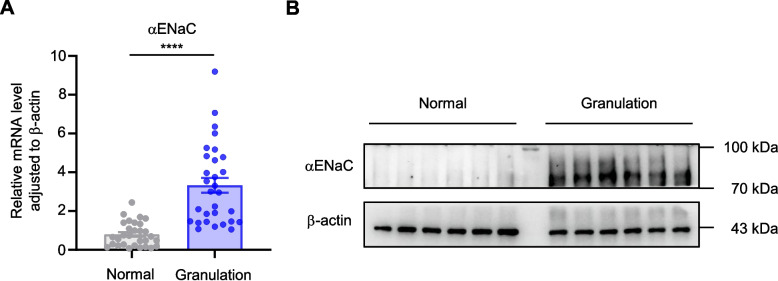
Significant upregulation of αENaC expression was detected in ERR granulation. **A** The mRNA expression of αENaC was measured in ERR granulation tissues (*n* = 30) and normal soft tissues (*n* = 30) via real-time PCR. **B** The protein expression of αENaC was measured in ERR granulation tissues (*n* = 6) and normal soft tissues (*n* = 6) via immunoblotting. Data were presented as mean ± SEM. Significance was expressed as: **** *p* < 0.0001

αENaC is expressed in many cancer cell lines and epithelial tissues, but its expression in granulation tissues has not been reported. Real-time PCR was used to determine the relative mRNA level of αENaC. αENaC was significantly upregulated in ERR granulation tissues taken between the two molars (*n* = 30) in comparison with the normal soft tissues located subalveolar (*n* = 30) (*p* < 0.0001) (Fig. 2A). As immunoreactive bands shown in Fig. 2B, αENaC presented higher concentrations in ERR granulation tissues (*n* = 6) on the protein level in comparison with normal soft tissues (*n* = 6).

### Correlation between αENaC expression and ERR degree of adjacent M2Ms

**Fig. 3:**
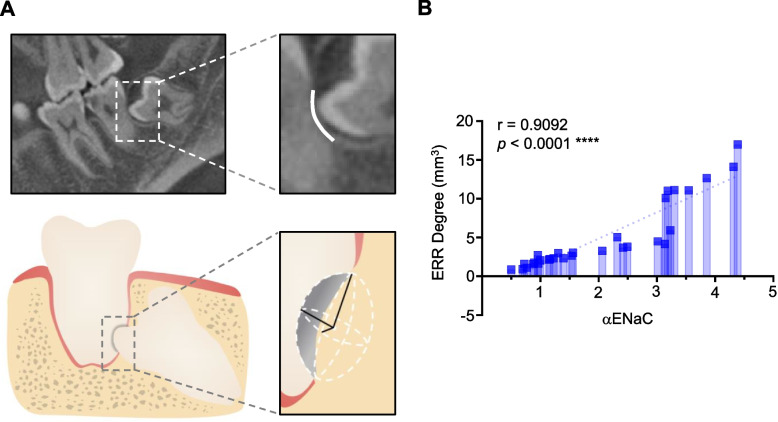
Upregulated αENaC was positively correlated with ERR degree of adjacent M2Ms in EIM3Ms. **A** Schema for the quantification of ERR degree with reference to a cone-beam tomography image. **B** Correlation between αENaC expression and ERR degree was evaluated via Spearman’s correlation analysis (*n* = 30). Data were presented as mean ± SEM. Significance was expressed as: **** *p* < 0.0001

From the schematic (Fig. 3A), we harmonized the quantification of ERR degree by approximating a resorption pit as a half of an ellipsoid. As shown in Fig. 3B, the quantified ERR degree was significantly positively correlated with αENaC in the ERR granulation tissues (*n* = 30, *r* = 0.9092, *p* < 0.0001), which demonstrated that αENaC aggravated ERR degree.

### Localization of αENaC to the fibroblasts and correlation between inflammation and EMT, and upregulated αENaC

**Fig. 4:**
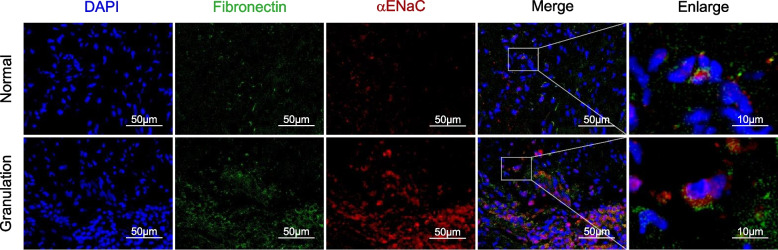
αENaC was localized to the fibroblasts in the ERR granulation tissue. αENaC (red), Fibronectin (green), nuclei (blue) were labelled via immunofluorescence in the ERR granulation tissue and the normal soft tissue. At higher magnification, there was significant colocalization of αENaC and Fibronectin in the ERR granulation. Scale bars: 50 μm and 10 μm (right)

The protein expression of αENaC was detected by immunofluorescence (Fig. 4). Evaluation of the ERR granulation tissue revealed markedly increased staining of αENaC in numerous cells, while feeble staining of αENaC was detected in the normal soft tissue. Interestingly, although both normal soft tissues and granulation tissues are composed of fibroblasts and their immunofluorescent staining of Fibronectin showed no significant difference, almost complete colocalization of αENaC and Fibronectin (the fibroblast marker) [21–23] staining was observed within the ERR granulation tissue, in contrast to the normal soft tissue located subalveolar, within which quite limited colocalization of αENaC and Fibronectin staining was showed. These analyses could illuminate that αENaC localized to the fibroblasts in the ERR granulation tissue.

**Fig. 5:**
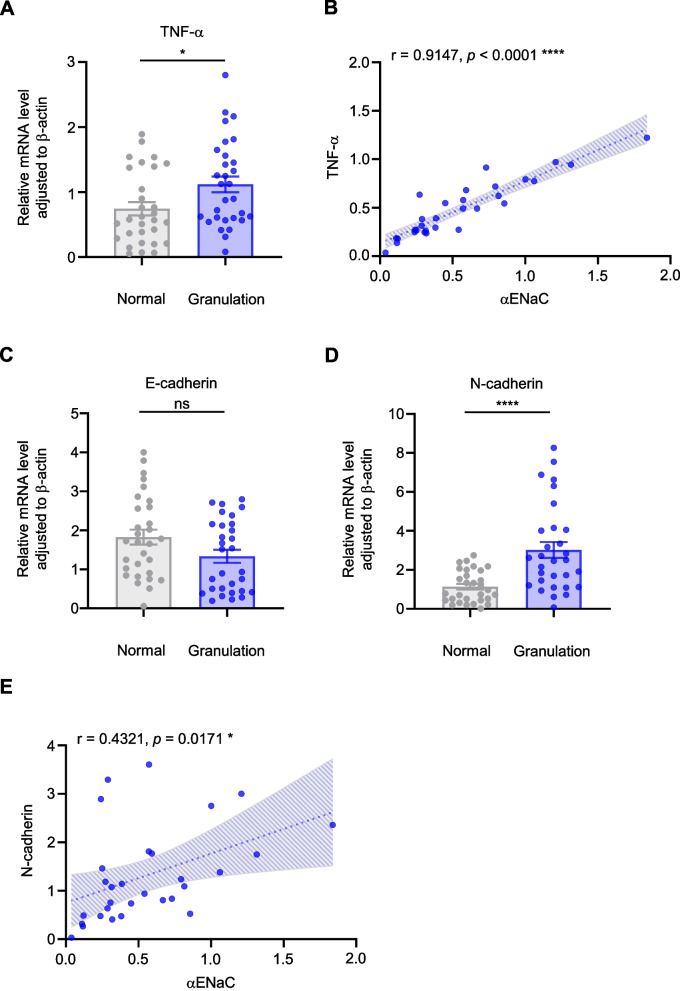
Upregulated αENaC was positively correlated with inflammation and epithelial-mesenchymal transition (EMT) in ERR from EIM3Ms. **A** The mRNA expression of TNF-α was measured in ERR granulation tissues (*n* = 30) and normal soft tissues (*n* = 30) via real-time PCR. **B** Correlation between αENaC expression and TNF-α was evaluated via Spearman’s correlation analysis (*n* = 30). **C**: The mRNA expression of E-cadherin was measured in ERR granulation tissues (*n* = 30) and normal soft tissues (*n* = 30) via real-time PCR. **D** The mRNA expression of N-cadherin was measured in ERR granulation tissues (*n* = 30) and normal soft tissues (*n* = 30) via real-time PCR. **E** Correlation between αENaC expression and N-cadherin was evaluated via Spearman’s correlation analysis (*n* = 30). Data were presented as mean ± SEM. Significance was expressed as: *p < 0.05 and **** *p* < 0.0001. ns, no significance

As shown in Fig. 5, TNF-α expression was significantly upregulated in ERR granulation tissues (*n* = 30) in comparison with normal soft tissues (*n* = 30) (*p* = 0.0199), and was significantly positively correlated with αENaC in ERR granulation tissues (*n* = 30, r = 0.9147, *p* < 0.0001). There was no significance of the E-cadherin expression in ERR granulation tissues (*n* = 30) and normal soft tissues (*n* = 30) (*p* = 0.0566), possibly because the both normal and granulation tissues included few epithelial cells. For N-cadherin, significant upregulation in ERR granulation tissues (*n* = 30) in comparison with normal soft tissues (*n* = 30) (*p* < 0.0001), and positive correlation with αENaC in ERR granulation tissues (*n* = 30, *r* = 0.4321, *p* = 0.0171) were shown. The involvement of αENaC might mediate the occurrence of inflammation and the transdifferentiation of periodontal epithelium residual into fibroblasts of ERR granulation tissue.

### Role of αENaC in the prevention of ERR due to EIM3Ms

**Fig. 6:**
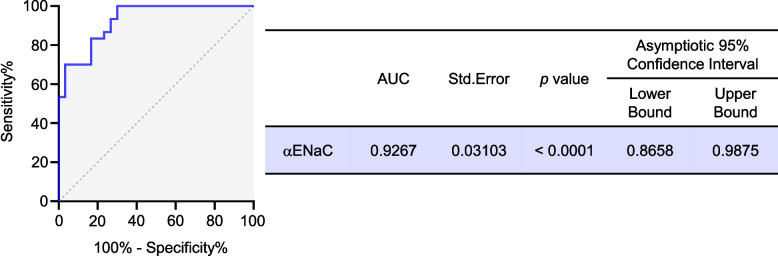
Receiver-operator characteristic (ROC) analysis of αENaC. The area under the curve (AUC) for αENaC was manifested in indicating the disease incidence (*n* = 30). Significance was expressed as: **** *p* < 0.0001

As mentioned above, αENaC was upregulated and positively correlated with ERR degree in granulation tissues of adjacent M2Ms due to EIM3Ms. Receiver-operator characteristic (ROC) curve was further manifested for αENaC to evaluate its capacity for indicating the incidence of the disease. As illustrated in Fig. 6, the area under the curve (AUC) was calculated as 0.9267 for αENaC (CI = [0.8658, 0.9875]). Reportedly, AUC takes values from 0 to 1, where a value of more than 0.9 suggests outstanding discrimination [24]. In consequence, our data illuminated that αENaC might be an effective indication of the incidence of the disease, aiming to prevent ERR of adjacent M2Ms due to EIM3Ms.

## Discussion

In our view, we revealed visually by FEA that ERR of M2Ms was caused by the force which EIM3Ms exerted on the distal root of M2Ms. αENaC was upregulated and positively correlated with the ERR degree in ERR granulation tissues. Immunofluorescence further demonstrated that αENaC localized to the fibroblasts in ERR granulation tissue. Moreover, αENaC was respectively and positively associated with TNF-α and N-cadherin, exerting effects on inflammation of ERR granulation tissues and EMT. More importantly, αENaC might be a novel indication of the incidence of this disease, for the purpose of preventing root resorption of adjacent M2Ms due to EIM3Ms.

Long-term impacted teeth are closely associated with ERR of adjacent teeth [1]. Clinically, it is illustrated that the force exerted on the distal root of adjacent M2M by an EIM3M results in ERR of the M2M. However, few have explored what happened there. Although the dental follicle of asymptomatic impacted mandibular third molars has been investigated from the radiological and histological view [25], few explored what happened in ERR of M2Ms due to EIM3Ms.

In this study, this clinical scenario is clearly visualized through the construction of a finite element model. From a mechanical perspective, we tested a mechano-sensitive channel there that no one has ever studied in this scenario. Here, we provided evidence that αENaC expression level was significantly elevated in ERR granulation tissues taken between two molars compared to normal soft tissues located subalveolar surrounding the roots, which was consistent with previous studies in cancer cells [26]. In addition, we quantified the ERR degree of M2Ms and found a positive correlation between ERR degree and αENaC expression in granulation tissues, which verified that αENaC could aggravate ERR degree of M2Ms.

Granulation tissue is mainly composed of capillaries, fibroblasts and inflammatory cells [27], and normal soft tissue (mainly periodontium) is mainly composed of fibroblasts, odontogenic osteoblasts, epithelial rests of Malassez, osteoblasts, osteoclasts and undifferentiated mesenchymal cells [28]. Although both tissues were composed of fibroblasts, we found αENaC was localized to the fibroblasts in the ERR granulation tissue. The epithelial remnant, also known as the Malassez epithelial remnant, is seen in the fibrous spaces in the periodontium adjacent to the root surface and is aligned parallel to the root surface [29]. The epithelial remnant, which is normally quiescent, can proliferate into jaw cysts and odontogenic tumors when stimulated by inflammation [30]. We therefore hypothesized that αENaC might play a role in regulating the process by which the epithelial remnants in the periodontium of the distal roots of M2Ms were transdifferentiated into the fibroblasts of the ERR granulation tissues taken between two molars.

Our findings then demonstrated that elevated αENaC was positively correlated with the upregulated expression of TNF-α (the relevant inflammatory factor) and N-cadherin (an EMT-related marker) in ERR granulation tissues. TNF-α has been proved to promote the mechanical force-induced ERR during orthodontic tooth movement [19], and important for osteoclast and odontoclast formation under compressive force [18]. Thus, αENaC promoted the root resorption with the involvement of TNF-α. As a common pro-inflammatory factor in granulation tissue, TNF-α can cause EMT in several types of cancer cells and epithelial cells [31–34]. It has been demonstrated that TNF-α activates NF-κΒ signaling pathway, and NF-κΒ activation induces EMT [35, 36]. NF-κΒ signaling pathway can promote osteoclast differentiation [37], and is crucial to TNF-α-induced EMT [38], which has been proven by the evidence that TNF-α-induced EMT can be inhibited by drugs that suppresses NF-κΒ activation [39]. Moreover, αENaC can be regulated by NF-κΒ [40]. Therefore, we speculated that αENaC might first promote TNF-α, and then TNF-α might further promote αENaC and EMT. More in-depth relevance of TNF-α, EMT and αENaC is worthy of further explorations.

In conjunction with the above, the ROC curve was performed for αENaC to evaluate its ability in indicating the incidence of the disease. According to excellent discrimination assessed by AUC value, αENaC might be a novel indication of the incidence of this disease. Early inhibition of αENaC expression may be able to prevent ERR to some extent, which requires to be further investigated.

## Conclusion

In summary, we searched for a new scenario in which the force exerted on the root of adjacent M2M by an EIM3M led to root resorption of the adjacent tooth. Our results also revealed the expression of αENaC in this scenario, and identified its localization and positive correlation with inflammation, EMT and disease severity. In addition, a potential ability of αENaC was verified for indicating the incidence of this disease. Our research put the spotlight on a new disease scenario and its mechanical mechanism and provided a new insight into the diagnosis and prevention of this disease relevant to EIM3Ms.

### Supplementary Information

**Supplementary Material 1.**

**Supplementary Material 2.**

## Data Availability

Data will be available from corresponding authors on the reasonable request.

## Abbreviations

EIM3Ms: Entirely impacted mandibular third molars
M2Ms: Mandibular second molars
ENaC: Epithelium sodium channel
Po: Opening probability
EMT: Epithelial-mesenchymal transition
3D: Three-dimensional
PCR: Polymerase chain reaction
EMR: Epithelial rest of Malassez
AUC: Area under the curve
ROC: Receiver-operator characteristic
