# Supplementary material for: Role of αENaC in root resorption of adjacent teeth due to entirely impacted mandibular third molars
Source: BMC Oral Health. 2024 Mar 21;24:360. doi: 10.1186/s12903-024-04040-z (PMC10956368; doi:10.1186/s12903-024-04040-z)
Supplement: Supplementary file 1 — Supplementary Material 1. [file 12903_2024_4040_MOESM1_ESM.docx]

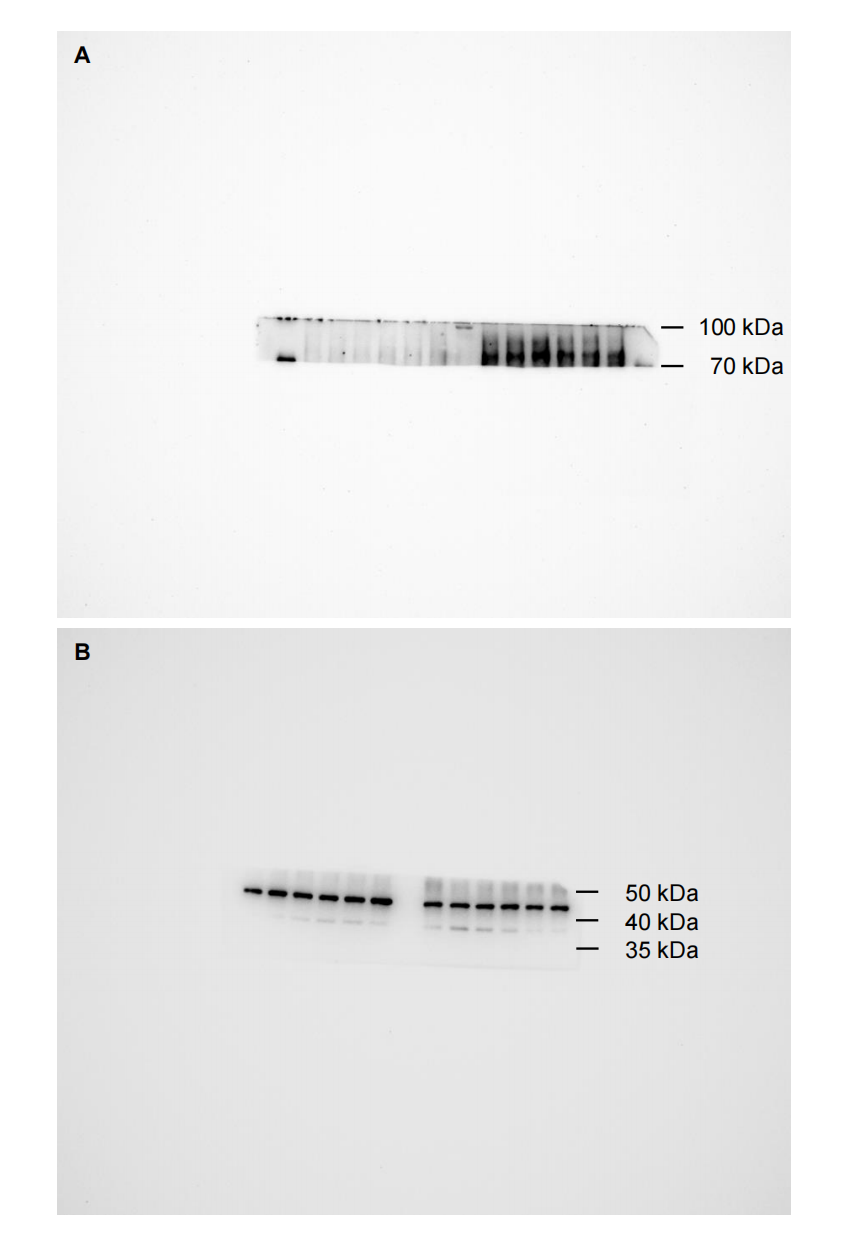


**Supplementary figure 1.** **A**: The protein expression of αENaC was measured in ERR granulation tissues (n=6) and normal soft tissues (n=6) via immunoblotting. **B**: The protein expression of β-actin was measured in ERR granulation tissues (n=6) and normal soft tissues (n=6) via immunoblotting.
